# Trends in Sugar-Sweetened Beverages: Are Public Health and the Market Aligned or in Conflict?

**DOI:** 10.3390/nu7095390

**Published:** 2015-09-23

**Authors:** William Shrapnel

**Affiliations:** Shrapnel Nutrition Consulting Pty Ltd., 790 Pinnacle Road, Orange, New South Wales 2800, Australia; E-Mail: shrapnelnc@bigpond.com; Tel.: +61-2-6365-3677

**Keywords:** sugar-sweetened beverages, trends, market failure, taxation, non-nutritive sweeteners, small changes

## Abstract

Adverse health consequences of consuming sugar-sweetened beverages are frequently cited as an example of market failure, justifying government intervention in the marketplace, usually in the form of taxation. However, declining sales of sugar-sweetened beverages in Australia and a corresponding increase in sales of drinks containing non-nutritive sweeteners, in the absence of significant government regulation, appear to reflect market forces at work. If so, the public health challenge in relation to sugar-sweetened beverages may have less to do with regulating the market and more to do with harnessing it. Contrary to assertions that consumers fail to appreciate the links between their choice of beverage and its health consequences, the health conscious consumer appears to be driving the changes taking place in the beverage market. With the capacity to meet consumer expectations for convenience and indulgence without unwanted kilojoules, drinks containing non-nutritive sweeteners enable the “small change” in health behaviour that individuals are willing to consider. Despite the low barriers involved in perpetuating the current trend of replacing sugar-sweetened beverages with drinks containing non-nutritive sweeteners, some public health advocates remain cautious about advocating this dietary change. In contrast, the barriers to taxation of sugar-sweetened beverages appear high.

## 1. Introduction

The consumption of sugar-sweetened beverages has been associated with increased risk for obesity and type 2 diabetes in the United States and lowering intake of these beverages is now a focus of public health nutrition in several countries [1,2]. Public health commentaries on the issue of sugar-sweetened beverages and health frequently position it as an example of market failure and recommend government intervention in the marketplace, usually in the form of taxation of these beverages [3]. So dominant is this view that other interpretations on the drivers of sugar-sweetened beverage consumption and viable, alternative strategies for lowering their use by the general public are seldom discussed.

This paper reviews a recent study that demonstrated a long-term downward trend in purchases of sugar-sweetened beverages in Australia and a corresponding uptrend in consumption of drinks containing non-nutritive sweeteners. Factors underpinning these trends, the barriers to their continuance and the relevance the prevailing public health paradigm for lowering intakes of sugar-sweetened beverages are considered.

## 2. A Trend Analysis of Water-Based Beverage Sales in Australia

A recent study investigated sales trends of non-alcoholic, water-based, ready-to-drink beverages in Australia for the period 1997–2011 [4]. The beverages included were carbonated soft drinks, sports drinks, energy drinks, iced tea, mineral water, mixers e.g., tonic water and ginger beer, and still water. These were categorised as either sugar-sweetened or non-sugar beverages. Data for fruit juice were not available. Annual national grocery volume sales data were sourced from AC Nielsen Scan Track and combined with foodservice, vending, convenience and dining purchase data imputed from beverage company research departments. These figures were then combined with data from a previous study [5] to provide a data set spanning 15 years from 1997 to 2011.

The volumes of sugar-sweetened beverages purchased fell by almost 11 litres per person during this period, while non-sugar beverages increased by almost 16 litres per person. Sugar-sweetened carbonated soft drinks declined from 75.8 to 56.1 litres per person between 1997 and 2011, a fall of almost 20 litres per person. Purchases of non-sugar carbonated soft drinks increased from 22.8 to 28.2 litres per person over the same period. Sales of still water increased by 12.4 litres per person. Over this 15-year period per capita sugar contribution of water-based beverages to the food supply fell from 9.2 kg to 7.6 kg. This was primarily due to falls in the sugar contributions from carbonated soft drinks, from 8.4 kg to 6.2 kg per person.

## 3. Discussion

The trends in sales of sugar-sweetened beverages observed in this study bring the current public health paradigm for addressing the health consequences of these drinks into question.

### 3.1. The Market Failure Argument

In considering public health strategies relating to sugar-sweetened beverages some authors have based their rationale on market failure, a scenario where a profit-driven industry meeting a consumer demand is having an adverse societal impact [3]. Several market failures relating to sugar-sweetened beverages have been outlined. Firstly, many consumers make the decision to consume sugar-sweetened beverages based on imperfect information *i.e.*, they fail to fully appreciate the links between their choice of beverage and its health consequences. A second failure relates to time-inconsistency, where impulsive behaviour results in a choice of beverage that would not have been made if it had been contemplated objectively and dispassionately. Thirdly, the market for sugar-sweetened beverages is associated with financial externalities *i.e.*, consumers do not bear the full costs of their decisions to buy a particular beverage as health costs are widely distributed across society through public and private health insurance schemes. In light of such market failures it has been argued that government intervention in the marketplace is warranted and fiscal regulation, or taxation, may be the preferred option [3]. Given the success of tobacco taxation in reducing the prevalence of smoking, the option of taxing of sugar-sweetened beverages has generated extensive debate [3,6,7,8,9,10] and was canvassed in a recent report by the World Health Organization [11].

The market failure argument may have appeared compelling in the context of the increases in consumption of sugar-sweetened beverages that occurred in the United States during the 1980s and 1990s [12,13] and the associated increase in the prevalence of overweight and obesity [14,15]. However, the upward trend in consumption of sugar-sweetened beverages has now been reversed in that country, with falls observed in children and adults since the early 2000s [16,17,18]. Two-thirds of the recent fall in intake of added sugars in the United States has resulted from decreased consumption of sugar-sweetened soft drink [19].

In light of the analysis of sales data of non-alcoholic, water-based, ready-to-drink beverages discussed above market failure as a rationale for taxation of sugar-sweetened beverages has even less resonance in Australia. This study identified a long-term decline in the proportion of this beverage category that was sugar-sweetened and corresponding increase in the proportion that was non-sugar. As a consequence, per capita sugar contribution from water-based beverages in Australia fell considerably between 1997 and 2011. Although these recent beverage trends in Australia and the United States are consistent with public health objectives they are occurring in the absence of significant taxation or government regulation of sugar-sweetened beverages, suggesting other factors are at play.

An alternative interpretation of recent beverage trends in both Australia and the United States is that they reflect market forces at work [20]. If so, the public health challenge may have less to do with regulating the market and more to do with harnessing it. Yet, hitherto, the factors driving the apparent replacement of sugar-sweetened beverages with non-sugar beverages have not been extensively assessed or debated in the scientific literature. Two likely key drivers of these trends are the health consciousness of consumers and the availability of acceptable alternatives to sugar-sweetened beverages.

### 3.2. The Health Conscious Consumer

Marketing literature is replete with information about the rising health consciousness of consumers, especially in relation to the maintenance of body weight [21]. According to Euromonitor, health and wellness have been major drivers of consumer purchasing decisions in the water-based beverage category in Australia [22] and the United States [23] in recent years, contradicting the assumption that consumers fail to appreciate the links between their beverage choice and its health consequences. While the challenge for beverage manufacturers is to translate this health consciousness into purchasing behaviour, their success can also coincide with beneficial public health outcomes. The key to success, from both perspectives, lies in closing the gap between the health aspirations of consumers and their beverage purchasing behaviour. Encouraging examples include recent brand launches of reduced-sugar or sugar-free products by major soft drink companies, their success in the marketplace likely being due to their capacity to meet consumer expectations for convenience and indulgence without unwanted kilojoules. In this context impulsive purchasing behaviour is not associated with significant costs to health.

### 3.3. Non-Nutritive Sweeteners: Facilitating the “Small Change”

Aspartame was approved for use in Australia in 1986 and its use in beverages coincides with the fall in purchases of sugar-sweetened drinks observed in the study discussed above. The availability of non-nutritive sweeteners with acceptable taste properties is likely to be the innovation that has enabled a significant proportion of consumers to switch from sugar-sweetened beverages to similar non-sugar alternatives. In relation to dietary strategies for weight management some authors have suggested a “small changes” approach on the basis that the advice will be seen by the general public to be more realistic, feasible to achieve and easier to maintain than advice to make large dietary changes [24]. The long-term beverage trends in Australia discussed above suggest that switching from a sugar-sweetened beverage to a similar-tasting, sugar-free drink is an example of the type of “small change” recommended by these authors. The efficacy of such a change is supported by two recent randomised controlled trials that showed the replacement of sugar-sweetened beverages with drinks containing non-nutritive sweeteners to be an effective weight control measure in children and adolescents [25,26].

Although there is a long-term up-trend in the sales of beverages containing non-nutritive sweeteners in Australia, sugar-sweetened drinks still dominate the category. Consumer concerns about the safety of non-nutritive sweeteners, fanned by social media commentary, may be a factor constraining the trend away from sugar-sweetened beverages. As the safety data on non-nutritive sweeteners are extensive, reassuring the population about their safety may be integral to public health initiatives to perpetuate current beverage trends. In a recent comprehensive re-evaluation of the safety of aspartame conducted by the European Food Safety Authority no issues of concern to the general population were identified and no changes were recommended to the maximum Acceptable Daily Intake [27]. The only exception to the recommended intake relates to people with phenylketonuria who are advised to avoid products sweetened with aspartame, though other non-nutritive sweeteners can be safely consumed by these individuals. Acesulphame K and stevia have also undergone extensive safety assessments by food regulatory authorities and are considered safe for use as general purpose, non-nutritive sweeteners by the general population [28,29]. Combinations of sweeteners are now employed to help meet the taste expectations of consumers.

On a more emotional plane, another factor constraining the shift from sugar-sweetened beverages to drinks containing non-nutritive sweeteners may be modern consumers’ desire for simplicity and naturalness in their foods, which has underpinned the rise in popularity of organic foods. Viewed through this prism sugar-sweetened beverages could be perceived to be more natural than drinks containing non-nutritive sweeteners. Against this background, Stevia, a plant extract rich in steviol glycosides, may satisfy consumers’ demand for naturalness in beverages while delivering the kilojoule benefit relative to sugar-sweetened beverages. Stevia has been approved for use in Japan for over three decades and is the major non-nutritive sweetener used in that country. Food Standards Australia New Zealand approved an application for stevia in 2008 and subsequently increased permitted levels in foods [29].

### 3.4. Proven Efficacy and Public Health Reticence

Despite the feasibility, sustainability, safety and efficacy of replacing sugar-sweetened beverages with drinks containing non-nutritive sweeteners, some public health advocates remain cautious about advocating this dietary change. Partly, this may be attributed to conservative interpretations of available evidence. One concern has been the observation that the consumption of beverages containing non-nutritive sweeteners has been associated with weight gain or increase in waist circumference in some prospective cohort studies [30,31,32,33]. It has also been suggested that the inconsistency between the intense sweet taste of non-nutritive sweeteners and the lack of associated energy consumed may lead to dysregulation of appetite control and, consequently, weight gain [34]. This has led to suggestions that drinks containing non-nutritive sweeteners may be fuelling the obesity epidemic rather than ameliorating it as could be expected. However, the cohort data need to be interpreted cautiously as reverse causality may be at play *i.e.*, those at higher risk of weight gain may be choosing to consume beverages containing non-nutritive sweeteners in an attempt to control weight. The results of the randomised controlled trials are reassuring in this regard [25,26,35].

Another factor limiting public health advocacy for the replacement of sugar-sweetened beverages with drinks containing non-nutritive sweeteners may be the tendency of health authorities to support optimal dietary outcomes. The Australian Dietary Guidelines (2013) and the recent evidence brief on sugar-sweetened beverages, obesity and health prepared by the Australian National Preventive Health Agency for the Australian Government both advocate the replacement of sugar-sweetened beverages with water [36,37]. This is undoubtedly the ideal exchange but in relation to the management or prevention of overweight there is not a strong evidence base for supporting the choice of water over a beverage containing a non-nutritive sweetener. Although it has been argued that the effects of consuming water or various other beverages remain under-studied [38], the results of a recent randomised trial suggest that beverages containing non-nutritive sweeteners are at least as efficacious as water when used as replacements for sugar-sweetened beverages [39]. The only other comparable trial produced similar findings [40]. In the Australian Dietary Guidelines the sole benefit of choosing water over beverages containing non-nutritive sweeteners was less dental erosion [36]. Advocacy for water consumption is to be encouraged and increasing sales of bottled water have been observed in Australia as sales of sugar-sweetened carbonated soft drinks have declined [4]. However, in the context of an obesity epidemic, nominating water as the only acceptable alternative to sugar-sweetened beverages appears to be a case of perfection being the enemy of good. All effective options need encouraged if optimal public health outcomes are to be achieved.

### 3.5. Taxation—The Path of Most Resistance?

In contrast to the low barriers involved in perpetuating the current trend of replacing sugar-sweetened beverages with drinks containing non-nutritive sweeteners, the barriers to taxation of the former appear high. The introduction of laws aimed at population-wide prevention of chronic diseases requires national political leadership at the highest level and broadly-based political support [41]. However, neither is in prospect in relation to the taxation of sugar-sweetened beverages. The idea of the state coercing its citizens to eat or drink in a particular way is anathema to libertarians who demand freedom from government interference and encourage personal responsibility [42]. When the left-of-centre opposition party in Tasmania recently lent support to the idea of taxing sugar-sweetened beverages it was immediately met with a “nanny state” accusation by the Minister for Health in that state [43]. Even in the scientific literature a well-reasoned argument in favour of taxation [3] was met with a letter underpinned by libertarian values [44]. Sharp political division over the use of taxation of food and beverages to achieve public health objectives is the likely explanation for why their implementation has hitherto been limited to France, Hungary, Finland, Mexico and Barbados. Assessments of public opinion about taxation of food and beverages to achieve public health ends conducted in the United States and United Kingdom have found low support [45,46]. Such measures were seen to be arbitrary, driven by the need to gather revenue rather than improve health, likely to disproportionately affect the poor and represent an unacceptable intrusion into people’s lives. Low trust in government was also a factor. Consumer concerns about the rationale for taxation appear to have been borne out by the introduction of taxes on both sugar-sweetened beverages and drinks sweetened with non-nutritive sweeteners in France in 2012. Whether beverage taxes lower sugar and kilojoule consumption in the long-term, improve the quality of national diets and have beneficial effects on public health have yet to be assessed in scientific studies. In the absence of widespread community support and with a stark political divide between the merits of market intervention and personal responsibility the likelihood that sugar-sweetened beverages will be subject to taxation in many countries appears remote [47].

## 4. Conclusions

Sugar-sweetened beverage consumption in Australia is in long-term decline, presumably driven by consumer concern about overweight, perceived links between sugar-sweetened beverage consumption and body weight and the availability of suitable alternative drinks that fulfill consumers’ expectations, such as bottled water and drinks containing non-nutritive sweeteners. Among those who currently consume carbonated beverages, the “small change” involved in moving from a sugar-sweetened beverage to a similar sugar-free beverage appears to be one that some consumers are willing to accept. Facilitating this change may be a more productive public health strategy than advocacy for taxation of sugar-sweetened beverages.
